# Urinary Arsenic in Human Samples from Areas Characterized by Natural or Anthropogenic Pollution in Italy

**DOI:** 10.3390/ijerph15020299

**Published:** 2018-02-09

**Authors:** Fabrizio Minichilli, Fabrizio Bianchi, Anna Maria Ronchi, Francesca Gorini, Elisa Bustaffa

**Affiliations:** 1 National Research Council—Institute of Clinical Physiology, 56100 Pisa, Italy; fabrizio.minichilli@ifc.cnr.it (F.M.); fabriepi@ifc.cnr.it (F.B.); francesca.gorini@gmail.com (F.G.); 2 Laboratory of Experimental and Clinical Toxicology, Maugeri Clinical Scientific Institutes, 27100 Pavia, Italy; anna.ronchi@fsm.it

**Keywords:** arsenic, epidemiology, biomarker, biomonitoring, urinary species

## Abstract

Arsenic is ubiquitous and has a potentially adverse impact on human health. We compared the distribution of concentrations of urinary inorganic arsenic plus methylated forms (uc(iAs+MMA+DMA)) in four Italian areas with other international studies, and we assessed the relationship between uc(iAs+MMA+DMA) and various exposure factors. We conducted a human biomonitoring study on 271 subjects (132 men) aged 20–44, randomly sampled and stratified by area, gender, and age. Data on environmental and occupational exposure and dietary habits were collected through a questionnaire. Arsenic was speciated using chromatographic separation and inductively coupled mass spectrometry. Associations between uc(iAs+MMA+DMA) and exposure factors were evaluated using the geometric mean ratio (GMR) with a 90% confidence interval by stepwise multiple regression analysis. The 95th percentile value of uc(iAs+MMA+DMA) for the whole sample (86.28 µg/L) was higher than other national studies worldwide. A statistical significant correlation was found between uc(iAs+MMA+DMA) and occupational exposure (GMR: 2.68 [1.79–4.00]), GSTT gene (GMR: 0.68 [0.52–0.80]), consumption of tap water (GMR: 1.35 [1.02–1.77]), seafood (GMR: 1.44 [1.11–1.88]), whole milk (GMR: 1.34 [1.04–1.73]), and fruit/vegetables (GMR: 1.37 [1.03–1.82]). This study demonstrated the utility of uc(iAs+MMA+DMA) as a biomarker to assess environmental exposure. In a public health context, this information could be used to support remedial action, to prevent individuals from being further exposed to environmental arsenic sources.

## 1. Introduction

Arsenic (As) is a naturally occurring element widely distributed throughout the earth’s crust, with higher concentrations in some geographical areas, in combination with either inorganic or organic substances that form many different compounds. Both organic As (oAs) and inorganic As (iAs) compounds can be emitted into the air, and then deposited into water and soil during industrial operations, such as ore mining and smelting, volcanic eruptions, and forest fires [1]. Arsenic and its inorganic compounds are classified as carcinogenic to humans [2,3]. Exposure to iAs in drinking water is associated with both carcinogenic and non-carcinogenic effects [3,4,5,6,7]. Despite As not being able to induce gene mutations [8,9,10], it can still be considered a genotoxic metalloid, as it induces micronuclei, DNA strand breaks, sister chromatid exchanges, chromosomal aberrations, and aneuploidy [11,12,13,14,15,16,17,18,19,20,21,22,23].

The main routes for human exposure to As are contaminated drinking water, contaminated water in food preparation and irrigation of food crops, industrial processes, contaminated food, and smoking tobacco [24]. Fish, shellfish, meat, milk, and cereals are the main contributors for As in the human diet, although As exposure from food is generally lower than from contaminated groundwater [25,26].

In drinking water, iAs is usually found in the form of arsenate [As(V)] and arsenite [As(III)]. The ingested iAs is mainly metabolized in the human liver [27]. The metabolic pathway consists in an alternation of reduction and oxidation reactions: reduction of the pentavalent form (As(V)) to form compounds such as monomethylarsonic acid (MMA(V)) and dimethylarsonic acid (DMA(V)), and oxidative methylation of (As(III)), monomethylarsonous acid (MMA(III)), and dimethylarsonous acid (DMA(III)) [28,29]. The metabolic pattern is considered a detoxification pattern because of the relatively low toxicity of MMA and DMA [30], therefore, As toxicity is closely related to its metabolism. Arsenic species in the trivalent state are generally considered more toxic at lower doses than the other As species [1,31]. However, recent investigations suggest that MMA(III) and DMA(III) are more toxic than iAs arsenite [32,33,34]. As the methylation process is incomplete, iAs along with MMA and DMA, are excreted in human urine. Vahter [35] showed that relative distributions of iA, MMA, and DMA in the urine of various populations are generally 10–30%, 10–20%, and 60–70%, respectively. On the other hand, there are large variations in As metabolism at individual and population levels [36]. It is known that biological and environmental factors, including age, sex, pregnancy, As exposure level, smoking habits, nutritional status, and diet, determine the inter-individual variations [37].

Compared with the convincing evidence on high-dose exposure, the risk assessment of exposures to low-to-moderate levels of environmental As is challenging for research and public health. The focus on the association between As exposure in drinking water in the concentration range of 10–100 µg/L and the health risk has increased, however, epidemiological studies are still limited, and results are not sufficiently consistent [38,39]. Several studies report associations between low doses of As and non-cancer diseases, such as cardiovascular diseases, diabetes, and neurological disorders, although epidemiological knowledge on this topic is limited or insufficient [40,41,42,43,44,45,46].

In Italy, the health risk to exposures to low-to-moderate As levels in drinking water is a great concern in many areas affected by As pollution of a natural and/or anthropogenic origin [47,48,49]. Directive 98/83/EC [50], in force since 2003, imposed the limit value of 10 µg/L for As in drinking water. Considering the specific geological conditions in different Italian regions that determine the natural occurrence of As in the aquifers used for the production of drinking water, Italy asked and obtained two derogations to 50 µg/L between 2003 and 2009, and a third one at 20 µg/L between 2010–2012 in four regions, including Tuscany and Latium (included in SEpiAs study) [51].

The health risk assessment for residents in As-contaminated areas is required in order to define primary prevention actions and to strengthen the control activities of public health through the development of an effective environment and health monitoring system, capable of providing rapid responses to administrators and citizens.

An epidemiological study in Italy called SEpiAs (Epidemiologic Surveillance in areas with natural or anthropic Arsenic pollution, funded by the Italian Ministry of Health), was thus carried out in four As-contaminated areas in Italy. The aim of SEpiAs, based on the Human BioMonitoring (HBM) of As and preclinical risks, was to assess the relationship between human As exposure, estimated by dose intake indicators, and biological markers of early health effects, in order to define indicators for an advanced environmental public health surveillance.

In the framework of SEpiAs, the distribution of urinary iAs (uiAs) plus methylated forms, such as urinary MMA (uMMA) and urinary DMA (uDMA), defined as u(iAs+MMA+DMA), was described.

The aims of this article are to

- compare the distribution of u(iAs+MMA+DMA) with other baseline international studies;
- assess the relationship between the u(iAs+MMA+DMA) concentration and various exposure factors investigated by the HBM questionnaire using stepwise multiple regression.

## 2. Materials and Methods

All detailed information on the materials and methods are provided in the SEpiAs report [49]. A summary is given of the study areas, samples, data collection, urine sample collection, As analysis, genetic susceptibility, and statistical methods.

Study areas—SEpiAs was carried out in two mountainous/hilly areas located in central Italy (Amiata in Tuscany, and Viterbese in Latium) and in two cities in southern Italy (Taranto in Apulia and Gela in Sicily). In Amiata and Viterbese, the As was of a natural origin (contamination of soil and water), while in Taranto and Gela, it was anthropogenic (contamination of soil, water, and air associated with industrial activities). The industrial areas of Taranto and Gela were declared as Reclamation Sites of National Interest on the basis of documented environmental contamination and/or presence of hazardous waste [48,52,53,54,55].

Study Sample—SEpiAs was designed as a multicentric epidemiological HBM-based sample survey. The initial objective was to study at least 200 unit samples (50 units for each area), however, during the preparation of the operative protocol, in order to meet local demand, 290 units was set as the objective. Of the 500 residents, randomly selected from the municipal registries, stratified by area, gender, and age (20–29, 30–39, 40–44 years), 341 subjects were contacted and invited to participate in the study. For each area and gender, the percentages of subjects sampled by the three age classes were 40%, 40%, and 20%, respectively. A total of 271 subjects were recruited (participation rate: 79.4% of the 341 contacted subjects, 93.4% of the initial objective of 290) (Table 1). A urine sample from these 271 subjects was also collected. Each subject was told not to consume fish for three days before the urine collection. This information was also checked by the questionnaire and by the organic uAs level.

Data collection—for the 271 subjects recruited, individual data on residential history, socio-economic status, environmental and occupational exposures, lifestyle, and dietary habits, were collected through a questionnaire. Variables considered in the literature associated with As concentration were selected, such as sex, age classes, area of residence, and education level. The following dichotomous variables were also selected, as they were considered principal pathways of As exposure [25,26]:

- usual consumption (more than twice a week) of tap water for drinking or cooking;
- usual consumption (more than twice a week) of meat, seafood, vegetables and fruit, bread, whole milk, coffee, wine, and beer;
- usual consumption (more than twice a week) of locally-produced meat, seafood, vegetables or fruit, bread and whole milk;
- occupational exposure in chemical industries and to industrial dust, chemical substances, gases, in particular: exposure to inorganic solvents and acids, oil derivatives, and silica (As is used for doping microchips);
- active cigarette smokers or former smokers who had given up less than six months ago;
- seafood consumption during three days before urine collection.

Urine Sample Collection and Arsenic Analysis—the choice of urinary biomarker depended on the source of As (natural or anthropogenic), and thus its chemical form. The most common biomarker is uAs, which reflects short term exposure to environmental and occupational sources of As [1]. uiAs and methylated species, such as uMMA and uDMA, were measured using a dynamic reaction cell inductively coupled plasma mass spectrometer (DRC ICP-MS), after chromatographic separation with HPLC (high-performance liquid chromatography). 

The validation process aims to demonstrate the validity of a method and the reliability of the results through the valuation of all parameters that are used (technical characteristics, applicability, analytical performance, etc.). The method limits of quantification for the different As species were in the range of 0.1–0.2 μg/L. Quantification was carried out by a seven point matrix-matched calibration in the range 0.1–20 μg/L. The accuracy for the determination of total As was tested by analyzing the quality control material Lyphochek 1 (urine metal control level 1, Bio-Rad, Irvine, CA, USA). The target value was 71 μg/L. Our average concentration from day to day (*n* = 20) was 70 μg/L (RSD = 6.5%), a value in very good agreement with the target value for total As [56]. As to MMA, DMA, As (III), and As (V), urine samples spiked with 20 μg/L of each As species, were analyzed (day-to-day, *n* = 20), and the average recovery was between 90% and 105%. Average intra- and inter-day repeatability, determined for total As and each As species, was <5%.

Concentrations below the limit of detection (LOD) of 0.2 μg/L, due to the instrument’s inability to detect extremely low levels of chemicals, were found in less than 10% of sampled subjects; a value of 0.141 (LOD/SQRT(2)) was assigned to measurements that were less than the LOD [57].

Genetic susceptibility—in order to define different metabolic and reparative capacities related to the genetic constitution, the presence of specific functional polymorphisms of genes involved in metabolic detoxification mechanisms was assessed in SEpiAs. This can create the basis for inter-individual differences in the triggering of biological effects and clinical factors related to As exposure. Genetic susceptibility was evaluated by a set of polymorphisms considered by the scientific literature to be associated with As methylation, such as AS3MT Met287Thr polymorphism in the arsenite methyltransferase gene (AS3MT) and glutathione *S*-transferase polymorphisms (GST-T1, GST-M1) [58,59,60].

Below, we report the characteristics of the new analyses that we performed in this study.

Urine biomarker—most studies on As speciation focus on the urine matrix, because collection is easy and because uAs is a good biomarker of recent exposure to iAs [61]. Arsenic and its metabolites are, in fact, rapidly absorbed in the intestine and 45–85% As is excreted in urine within 2–3 days after exposure [62]. uAs can also be used both to monitor subjects occupationally exposed and for population studies, especially in continuous exposures influenced by local anthropogenic factors. However, it can also be considered a good indicator for chronic exposure [63]. Total urine As (tuAs) minus arsenobetaine (AsB) has been used as a marker of iAs exposure in several studies [64,65,66,67].

DMA is the most abundant As species occurring in urine after exposure to iAs as a result of As metabolism [28]. Seafood, including fish, shellfish, and seaweed, are important sources of organic arsenicals (AB, arsenosugars, and arsenolipids), which are believed to have a low toxicity [68,69,70]. Seaweed, mollusks, and fatty fish are rich in arsenosugars and/or arsenolipids, which are metabolized into several As species, including DMA, dimethylated thio As species, and possibly MMA [71,72,73,74]. Therefore, in populations with a moderate-to-high fish intake, the sum of inorganic and methylated As species levels in urine is not considered as the best biomarker of iAs intake [75]. One study demonstrated increased tuAs metabolites, especially DMA, in a group of volunteers after consuming seaweed. Thus, uDMA does not represent occupational exposure to As, but is a marker of seafood intake [76].

In the absence of seafood intake, DMA accounts for ~60–80% of tAs in urine [28,77,78]. In populations with a low seafood intake, the sum of inorganic and methylated As species levels in urine correlates well with As intake from drinking water and dietary sources, and is an accepted biomarker of iAs exposure [48,79,80]. Hakala et al. (1995) [81] performed a study in copper smelter workers to assess occupational As exposure. They showed that uiAs (As^3+^, As^5+^) is more useful for assessing occupational exposure to As, than tuiAs metabolites. In this study, the concentration of iAs (As^3+^, As^5+^) was significantly different between groups [81]. Because seafood consumption in Asian countries is higher than in Western countries, on the basis of their findings in the Japanese general population, Hata et al. (1995) [82] recommended excluding DMA when assessing occupational exposure to As.

In SEpiAs, two regions were characterized by anthropogenic As pollution (Taranto and Gela) and two by natural As pollution (Viterbese and Amiata). Considering the heterogeneity of the exposure factors (diet, genetic susceptibility, occupational and environmental factors) among the four areas, we decided to use u(iAs+MMA+DMA) as the best biomarker for recent As exposure according to the scientific literature [1,48,83,84,85]. This way, all values would be comparable.

Statistical methods—the statistical analyses were performed using u(iAs+MMA+DMA). In order to generate a residual distribution close to a normal distribution, a logarithmic transformation of u(iAs+MMA+DMA) was carried out. To evaluate heterogeneity and variability, u(iAs+MMA+DMA) distribution by area and gender using geometric mean (GM), 5th, 25th, 50th, 75th, and 95th percentiles (5p, 25p, 50p, 75p, and 95p, respectively) and standard deviation (SD) were presented and discussed. The use of u(iAs+MMA+DMA) enables GM and 75p to be compared with results from other Italian and international studies. For the whole sample and for each area, factors influencing As levels were identified through multivariate regression with backward stepwise selection, starting from the selected factors and removing terms with a *p*-value ≥ 0.2. The associations between u(iAs+MMA+DMA) and each significant estimator were reported using the GM ratio (GMR), with a 90% confidence interval (90% CI), adjusted for the other significant factors. Statistical significance was set at *p* < 0.1. In the multivariate regression analysis, factors with few sample units (<3 subjects) by class of exposure were not considered. Multivariate regression analysis was performed on 267 subjects, because of a lack of genetic data for four subjects. In order to highlight associations similarities among exposure factors and inorganic/organic As species, separate analyses considering uiAs and u(MMA+DMA) were also performed for the complex and for each area. All the analyses were carried out using STATA 13 [86].

All subjects gave their informed consent for inclusion before they participated in the study. The study was conducted in accordance with the Declaration of Helsinki, and the protocol was approved by Ethics Committee of the provincial healthcare company of Viterbo, Caltanissetta (for Gela), Siena (for Amiata) and Taranto. Project Identification Code: B51J10001120005.

## 3. Results

### 3.1. Distribution of u(iAs+uMMA+uDMA) Levels by Area and Gender

The results obtained from validation process of As speciation showed their suitability for the study, and confirmed the high linearity, sensitivity, precision, and accuracy of the method used.

**Figure 1:**
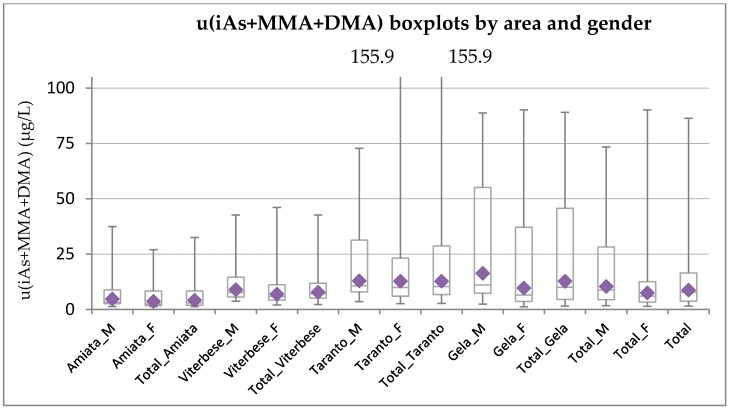
Distribution of u(iAs+MMA+DMA) (µg/L) by area and gender. Notes: Diamonds represent the GM; upper whiskers represent 95th percentile.

Figure 1 shows high heterogeneity among areas, high variability within areas, and various differences between genders. Taranto and Gela have a greater internal variability than Viterbese and Amiata.

Table 2 shows that Taranto and Gela had higher u(iAs+MMA+DMA) concentrations (Taranto: GM = 12.77 μg/L; Gela: GM = 12.68 μg/L) than Viterbese (GM = 7.73 μg/L) and Amiata (GM = 4.13 μg/L).

Higher concentrations were observed in men in each area, with a stronger difference in Gela (GM difference = 6.65 μg/L), and to a lesser extent in Viterbese (GM difference = 1.99 μg/L).

### 3.2. Stepwise Multiple Regression

Table 3, Table 4, Table 5, Table 6 and Table 7 show the results of the multivariate analysis of exposure factors on GM of u(iAs+MMA+DMA).

Total sample (Table 3) showed a significant difference in u(iAs+MMA+DMA) by area and a significant increase in GM according to GSTT polymorphism (presence of null genotype), consumption of seafood (adjusted for consumption the three days before urine collection), consumption of whole milk, wine, meat, whole milk, fruit, and vegetables of own/local production (all estimates difference were adjusted for the other factors).

We observed a statistically significant difference (*p* < 0.001) among GMs of the four areas with Taranto and Gela showing higher values, 11.75 µg/L and 13.42 µg/L, respectively, compared to Viterbese and Amiata, 8.60 µg/L and 3.86 µg/L, respectively. A statistically significant decrease (*p* = 0.014) in the GMs of u(iAs+MMA+DMA) concentration was observed among GSTT positive genotype carriers (8.12 vs. 12.02 µg/L). Subjects occupationally exposed to chemical industrials had higher GM values than those not exposed (21.87 vs. 8.17 µg/L) (*p* < 0.001). Seafood consumption (both in general and three days before urine collection) was also a factor associated with u(iAs+MMA+DMA) concentration (*p* = 0.023). In fact, subjects that consumed seafood had u(iAs+MMA+DMA) higher GM values than those who do not consume fish (9.57 vs. 6.62 µg/L), and also, if the seafood is consumed three days before urine collection (13.66 vs. 7.75 µg/L) (*p* < 0.001). A statistically significant increase in GM concentration was also observed among those consuming whole milk of own/local production (*p* = 0.049) and not (*p* = 0.057) (11.03 vs. 8.06 µg/L and 11.06 vs. 8.25 µg/L, respectively). No significant increase in GM values was observed among subjects consuming wine (*p* = 0.155) and meat (*p* = 0.113).

Significant exposure factors identified by the analyses on uc(iAs) are the same as those identified by the analyses on uc(MMA+DMA). These results are in line with those obtained with analysis performed with uc(iAs+MMA+DMA). Complete results of the analyses carried out for the overall sample are reported in the Tables S1 and S2.

From the multivariate regression analyses, in Amiata we found a statistically significant (*p* = 0.05) decrease in the GM of u(iAs+MMA+DMA) concentration among carriers of the GSTT positive genotype (3.21 vs. 5.57 µg/L). Significant increases (*p* = 0.075) in GM for u(iAs+MMA+DMA) concentration were also observed for smokers versus non-smokers (4.97 vs. 3.10 µg/L) and among consumers of seafood three days before the urine collection (6.56 vs. 3.02 µg/L) (*p* = 0.007), meat (4.62 vs. 2.85 µg/L) (*p* = 0.056), and whole milk of own/local production (9.17 vs. 3.51 µg/L) (*p* = 0.011) (Table 4).

The Viterbese sample showed a significant increase (*p* = 0.076) in GM in subjects drinking tap water or using tap water to cook with (9.23 vs. 6.85 µg/L) (Table 5). Statistically significant increases in GM were also found for smokers (10.55 vs. 7.50 µg/L) (*p* = 0.046), for consumers of whole milk (10.75 vs. 7.77 µg/L) (*p* = 0.061), and seafood in the three days before the urine collection (19.75 vs. 6.46 µg/L) (*p* < 0.001). A statistically significant increase (*p* = 0.016) in GM value of u(iAs+MMA+DMA) concentration was observed among subjects occupationally exposed to inorganic solvents and acids compared with those not exposed (19.42 vs. 6.90 µg/L) (Table 5).

The Taranto sample showed a significant increase in GM values in consumers, compared to non-consumers, of some foodstuffs, such as seafood (7.34 vs. 3.56 µg/L) (*p* = 0.044), meat (8.31 vs. 4.78 µg/L) (*p* = 0.06), bread, pasta, and cereals (6.61 vs. 1.85 µg/L) (*p* = 0.027), whole milk (14.75 vs. 4.81 µg/L) (*p* < 0.001), coffee (7.64 vs. 3.43 µg/L) (*p* = 0.018), and fruit/vegetables of own/local production (11.17 vs. 4.95 µg/L) (*p* = 0.015). A statistically significant increase (*p* = 0.002) in GM concentration was observed among subjects occupationally exposed to inorganic solvents and acids, compared with the non-exposed (19.42 vs. 6.90 µg/L). No significant increase in GM values was observed among subjects with AS3MT polymorphism (presence of Met287Thr) (*p* = 0.18) and consuming tap water (*p* = 0.105) (Table 6).

The Gela sample showed a significant increase in GM of u(iAs+MMA+DMA) concentrations in males exposed to occupational factors in the chemical industry (36.67 vs. 10.48 µg/L) (*p* < 0.001), and among seafood consumers compared to non-consumers (13.37 vs. 6.89 µg/L) (*p* = 0.023) (Table 7). We found a statistically significant decrease in GM concentration both among carriers of the null genotype of GSTT (9.68 vs. 22.43 µg/L) (*p* = 0.008) and among those drinking coffee (9.81 vs. 19.86 µg/L) (*p* = 0.012).

## 4. Discussion

The aim of our research was to compare the distribution of u(iAs+MMA+DMA) in people living in four Italian areas, with other baseline international studies, and to assess the relationship between u(iAs+MMA+DMA) concentration and various exposure factors investigated through a specific HBM questionnaire.

Regarding the first objective, the results obtained from descriptive analyses suggest that the As exposure for inhabitants in industrial areas with high environmental pressure was greater than for those living in areas with natural contamination (Table 2). These results are in line with other studies. Janasik et al. reported a correlation between occupational iAs pollution and both u(iAs+MMA) (statistically significant) and u(iAs+MMA+DMA) (not statistically significant) [87]. Two other studies found a correlation between occupational exposure and u(iAs+MMA+DMA) [81,88]. To explain the high variability within the areas, the presence of multifactorial exposures must be taken into account. The sampled subjects have different lifestyles, eating habits, professional exposure, and genetic characteristics. Furthermore, it can be reasonably assumed that the interaction between these factors may have an effect on the inter-subject variability.

The observed 95p value of 86.28 µg/L for u(iAs+MMA+DMA) for all areas and for both genders, is higher than the value defined by the Italian Society of Reference Values (ISRV) of 15 µg/L [89]. We also compared our values with the results of studies (Table 8) conducted in Germany [90,91], France [83,92], and in the United States [57]. All SEpiAs GMs, median, and 95p values were significantly higher than the references (Table 8). Considering the individual areas, only Amiata presented a lower GM than the values reported in the other studies (Table 8).

Our data were also compared with those observed in a similar study conducted in Mexico. The Mexican study was carried out in four towns (43 subjects) in the Yaqui Valley, in order to characterize uAs excretion among adults. The GM values for u(iAs+MMA+DMA) concentrations in the selected towns ranged from 28.0 µg/L to 65.1 µg/L [93]. The lower value of this range was higher than the GM value (8.76 µg/L) for u(iAs+MMA+DMA) observed in our study.

A recent study conducted in Taranto found a mean value of 6.1 µg/L for u(iAs+MMA+DMA) [94], which is lower than the value we observed of 27.36 µg/L.

In Inner Mongolia, in an endemic area for As poisoning where the groundwater typically contains high As concentrations of up to 1354 μg/L, with a mean value of 173 µg/L, the mean u(iAs+MMA+DMA) concentration was 300.17 µg/L [30]. In the same area, another study found mean u(iAs+MMA+DMA) concentrations of 252.84 µg/L [95]. The values observed in SEpiAs are lower than the values observed in Mongolia. This is due to the fact that Mongolia is characterized by high As concentrations in the groundwater.

Regarding the second aim of this study, the results are discussed below for each statistically significant exposure factor identified by our analyses.

Occupational exposure—we observed a statistically significant correlation between u(iAs+MMA+DMA) concentration and occupational exposure in the overall sample, and in Taranto and Viterbese. Our findings are consistent with previous results. A recent study conducted in Poland on 149 workers in a copper mill showed a statistically significant correlation between iAs concentrations in the air and u(iAs+MMA) concentration [87]. The same correlation was reported considering u(iAs+MMA+DMA) concentration, although not statistically significant. This was probably the result of increased DMA concentrations, especially in individuals who confirmed that they had eaten fish dishes before the study [87]. A study carried out in Manfredonia, in Apulia in southern Italy, highlighted that the mean values of u(iAs+MMA+DMA) were 23.9 µg/L in July 2006 and 15.1 µg/L in August–October 2006, before and after a recommended more careful use of personal protective equipment, respectively [96].

Tap water—a statistically significant association between u(iAs+MMA+DMA) concentration and tap water consumption was observed in the Viterbese and Taranto samples. Other studies found the same association. A study conducted in Pabna, Bangladesh, investigating factors influencing biomarkers of As exposure, reported that u(iAs+MMA+DMA) concentrations were significantly associated with As concentration in drinking water (*p* < 0.001) [97]. In a study performed in inner Mongolia among inhabitants of a rural area, Wei et al., showed a positive association between As in drinking water and u(iAs+MMA+DMA) [30]. In a recent study carried out in Taranto [94], the authors reported higher median values of u(iAs+MMA+DMA) in those who drank tap water (3.6 µg/L) than those who drank bottled mineral water (2.5 µg/L).

Seafood consumption—we observed an overall statistically significant association between u(iAs+MMA+DMA) and both seafood consumption and seafood consumption three days before the sampling. Considering other national surveys, the German study found that the frequency of fish consumption was the most dominant determinant [90,91]. Saoudi et al., observed that concentrations of tuAs and u(iAS+MMA+DMA) were influenced by sociodemographic and economic factors, and by risk factors such as consumption of seafood products and wine [83]. A study involving participants of NHANES 2003–2006 evaluated the association of seafood intake with spot uAs concentrations [79]. Although different biomarkers were used compared with those of our study, their study found that participants reporting any seafood intake in the past 24 h had increased levels of all uAs biomarkers than participants with no seafood intake [79], thus concluding that seafood intake was a key determinant of increased urine concentration of some As metabolites [79].

A cross-sectional study was carried out in France, to evaluate As exposure of residents living in an area with a soil naturally rich in As, through urinary measurements. Significant associations were found between u(iAs+MMA+DMA) concentration and consumption of seafood (*p* = 0.03), consumption of wine (*p* = 0.03), and beer (*p* = 0.001), respectively three and four days before the investigation [98]. These findings were also confirmed by a Korean study that reported a significantly positive relationship between As intake from diet and u(iAs+MMA+DMA) concentration. This finding suggested that dietary As intake may affect the total As levels in urine. Further analyses were performed to assess the correlation of urine As with the consumption of specific food groups. In particular, u(iAs+MMA+DMA) concentration was positively correlated with the consumptions of specific food groups, such as seaweed (*p* < 0.01), fish and shellfish, and grains, however, it was negatively correlated with meat consumption [99].

Considering individual areas, we found that u(iAs+MMA+DMA) concentrations were associated with fish consumption in Taranto and Gela, and with seafood consumption three days before the sampling in Amiata and in Viterbese. An Italian study conducted in Manfredonia (Apulia), showed a statistical association (*p* < 0.001) only between the consumption of fish and shellfish 48–72 h prior to urinary sampling and the excretion of u(iAs+MMA+DMA). Comparing the levels of excretion of iAs and its methylated metabolites between the two monitoring phases (July 2006 and August–October 2006), there was a statistically significant reduction in the investigated biomarker [96]. Another Italian study carried out in the provinces of Viterbo, Rome, and Latina assessed iAs exposure and metabolism in 269 residents from 27 municipalities [48]. The u(iAs+MMA+DMA) concentration in subjects using water with As levels >10 µg/L was taken as an estimate of the iAs historical exposure of the population. The average concentration exceeded the upper limit of the reference concentration range of 2–15 μg/L proposed for the Italian population [89], and most individual levels were also above the upper limit. In the study conducted in Taranto by Vimercati et al., statistically significant differences were shown when comparing u(iAs+MMA+DMA) concentrations in consumers of shellfish and/or seafood in the 48–72 h before sampling (9.8 vs. 3.8 μg/L) [94].

Genetic factors: GSTT—after the multivariate regression analysis, we found a statistically significant decrease in the GM of u(iAs+MMA+DMA) concentration according to carriers of the GSTT positive genotype. Considering individual areas, the same results were found in Amiata and Taranto. These findings are consistent with a study in Chile, in which 66 subjects from Antofagasta who were exposed to low levels of As were examined, in order to evaluate the relationship between polymorphic variants of GST and As species urinary concentration [100]. After adjusting As species level for creatinine, the absolute level of u(iAs+MMA+DMA) was higher among null GSTT1 carriers than among those carrying the active gene (*p* = 0.062) [100], as reported in our study, despite the lack of creatinine adjustment. In Vietnam, a study investigated the association of genetic polymorphisms in the members of glutathione *S*-transferase (GST) superfamily with As levels in hair and urine, and the uAs profile in residents in the Red River Delta [101]. Arsenic concentrations were given as single metabolites As(III), As(V), DMA(V), MMA(V), and arsenobetaine, As(III) + As(V), and the overall sum and u(iAs+MMA+DMA) were not reported. No significant associations between GSTT1 wild/null with concentrations and compositions of uAs were found [101]. In a study conducted in Bangladesh [94], GSTT1 was slightly associated with increased utAs reported as u(iAs+MMA+DMA) (*p* = 0.06) in adjusted models. In particular, individuals with the null genotype had a slightly higher excretion rate of As, compared to individuals with a wildtype GSTT1 genotype, after adjusting for some factors (including creatinine). The study by Kile et al., confirmed our findings, thus, it is likely that GSTT influenced the relative concentration of methylated uAs metabolites.

This study presents some limitations. The results are based on a single urine sample from each participant. Hence, we could not establish the reproducibility and accuracy of the measurements. Instead of asking participants to avoid seafood consumption within three days before the urine collection, through a questionnaire we asked if they had consumed seafood, and used this variable to adjust the analyses. 

Despite these limitations, this study allowed us to examine, in depth, the relationships between uiAs plus uAs methylated species in subjects living in areas with recognized low-to-moderate concentrations of As pollution of an anthropogenic or natural origin.

Our results highlighted considerable differences among the four areas and between industrial and natural polluted areas, as previously reported in the literature [81,87,88]. In fact, a comparison between areas with natural or anthropogenic As pollution and reference areas, suggested that As contamination led to higher u(iAs+MMA+DMA) in the industrial areas of Gela and Taranto. These findings highlight the heterogeneous profiles among areas, with a marked difference between Amiata and Gela, similarities between males and females within the samples of Amiata and Viterbese and, conversely, differences by gender within the samples of Gela and Taranto.

There is wide evidence demonstrating that some individuals are more susceptible to As [102,103], and genetic factors can also play a role in susceptibility [104,105]. The factors we found to be associated with the urinary profile of As are supported by the scientific literature: consumption of seafood [48,79,83,90,91,94,96,98,99], consumption of contaminated tap water [30,94,97], occupational exposure [87,96], and some polymorphisms [97,100,101]. We also identified some other factors, such as wine, grains, meat, and milk consumption, which make for interesting further investigations.

In our study, the factors commonly reported in the literature as associated with the organic or inorganic arsenic forms are associated with both inorganic and organic forms, considered separately and as a sum. Therefore, we suggest the use of uc(iAs+MMA+DMA) biomarker in studies of areas with different exposure pathways.

## 5. Conclusions

Due to the widespread presence of arsenic in the environment and its potential impact on health, the health system is often called into question. Our results highlight higher arsenic exposure in areas characterized by natural or anthropogenic arsenic pollution compared to national and international reference areas. The study therefore confirms the need for an environmental and health surveillance system in recognized areas with documented contamination.

Our results confirm that occupational exposure, and consumption of tap water and fish, represent the main factors of exposure. The study also highlights the role of genetic susceptibility and indicates the need to study further exposure factors, such as the consumption of meat, milk, fruit, and vegetables. With the appropriate sample collection, analysis, and interpretation, biomonitoring in conjunction with questionnaires can provide an accurate picture of environmental exposure.

Considering the significant differences in sources of exposure, biotransformation in the human body, and toxicity of inorganic and organic forms, our results highlight the need for arsenic speciation in appropriate arsenic risk assessment and together with the utility of u(iAs+MMA+DMA) as a biomarker in areas with different exposure pathways.

In a public health context, this information could be used to support remediation measures to reduce exposure to arsenic. Recent advances in genomics and epigenetics offer additional insight into the toxicity of arsenic and into the mechanisms of arsenic carcinogenicity. Identifying polymorphisms, gene–environment interactions, and related effects on arsenic metabolism, will provide important information on the mechanisms behind the biotransformation of arsenic, and also facilitate comprehension of individual differences in arsenic metabolism. This will help us to identify susceptible groups, and may provide better risk estimates for arsenic.

## Figures and Tables

**Table 1:** Distribution by area, gender, and age (20–29, 30–39, 40–44 years) of the 271 subjects recruited after the accession to the interview and the collection of a urine sample. The percentage was calculated on the contacted subjects by area.

Area	Male				Female				Total			
	20–29 (%)	30–39 (%)	40–44 (%)	Total (%)	20–29 (%)	30–39 (%)	40–44 (%)	Total (%)	20–29 (%)	30–39 (%)	40–44 (%)	Total (%)
Amiata	10 (62.5)	12 (75.0)	6 (75.0)	28 (70.0)	11 (68.7)	11 (68.7)	8 (100.0)	30 (75.0)	21 (65.6)	23 (71.9)	14 (87.5)	58 (72.5)
Viterbese	15 (88.2)	11 (68.8)	6 (66.7)	32 (76.2)	16 (94.1)	15 (93.8)	9 (100.0)	40 (95.2)	31 (91.2)	26 (81.3)	15 (83.3)	72 (85.7)
Taranto	11 (84.6)	9 (69.2)	4 (66.7)	24 (75.0)	11 (84.6)	10 (76.9)	5 (83.3)	26 (81.3)	22 (84.6)	19 (73.1)	9 (75.0)	50 (78.1)
Gela	16 (69.6)	20 (86.9)	12 (100.0)	48 (82.8)	23 (100.0)	12 (52.2)	8 (66.7)	43 (74.1)	39 (84.8)	32 (69.6)	20 (83.3)	91 (78.4)
Total	52 (75.4)	52 (76.5)	28 (80.0)	132 (75.9)	61 (88.4)	48 (70.6)	30 (85.7)	139 (80.8)	113 (81.9)	100 (73.5)	58 (82.6)	271 (78.8)

**Table 2:** Descriptive analysis on u(iAs+MMA+DMA) (µg/L) by area and gender.

Statistics	Amiata			Viterbese			Taranto			Gela			Total		
	M	F	M + F	M	F	M + F	M	F	M + F	M	F	M + F	M	F	M + F
*n*	28	30	58	32	40	72	24	26	50	48	42	91	132	139	271
AM	10.23	6.24	8.16	12.89	13.37	13.15	24.24	30.23	27.36	29.89	24.76	27.47	20.57	18.51	19.51
GM	4.70	3.66	4.13	8.90	6.91	7.73	12.87	12.68	12.77	16.29	9.64	12.68	10.35	7.47	8.76
SD	19.54	7.96	14.73	12.18	28.55	22.64	34.34	56.57	46.86	30.07	32.96	31.26	26.77	35.03	31.24
5p	1.30	1.32	1.26	3.67	1.99	2.23	3.54	2.62	2.72	2.36	1.19	1.46	1.73	1.42	1.50
25p	2.65	1.75	1.92	5.58	4.20	5.15	7.92	5.93	6.64	7.23	3.58	4.51	4.42	3.27	3.71
50p	3.22	2.42	3.02	7.71	5.80	7.05	10.69	9.91	10.25	10.96	6.52	9.95	8.81	5.99	7.78
75p	8.78	8.27	8.27	14.50	11.07	11.83	31.33	23.15	28.68	55.07	37.12	45.65	28.21	12.47	16.37
95p	37.37	26.92	32.49	42.58	46.04	42.58	72.79	155.91	155.91	88.68	90.08	89.07	73.37	90.08	86.28

Notes: *n*: sample size; AM: arithmetic mean; GM: geometric mean; SD: standard deviation; 5p: 5th percentile; 25p: 25th percentile; 50p: 50th percentile; 75p: 75th percentile; 95p: 95th percentile M: males; F: females.

**Table 3:** Overall sample. Factors associated with u(iAs+MMA+DMA) concentration by stepwise multivariate regression analysis.

Factors Selected (*p* < 0.2)	Class	GM Exp.	90% CI	GMR	90% CI
Area	Amiata	3.86	3.05–4.89	1 (reference)	
	Viterbese	8.60	6.93–10.68	2.23	1.62–3.06
	Taranto	11.75	8.82–15.66	3.05	2.08–4.46
	Gela	13.42	11.02–16.34	3.48	2.54–4.76
GSTT	-	12.02	9.56–15.13	1 (reference)	
	+	8.12	7.21–9.15	0.68	0.52–0.88
Occupational exposure in chemical industrials	No	8.17	7.31–9.12	1 (reference)	
	Yes	21.87	14.92–32.05	2.68	1.79–4.00
Seafood	No	6.62	5.24–8.37	1 (reference)	
	Yes	9.57	8.49–10.78	1.44	1.11–1.88
Seafood consumption 3 days before urine collection	No	7.75	6.86–8.75	1 (reference)	
	Yes	13.66	10.95–17.03	1.76	1.37–2.27
Whole milk	No	8.25	7.30–9.31	1 (reference)	
	Yes	11.06	8.88–13.77	1.34	1.04–1.73
Wine	No	8.69	7.80–9.67	1 (reference)	
	Yes	13.88	8.17–23.58	1.60	0.93–2.75
Meat	No	7.96	6.83–9.27	1 (reference)	
	Yes	9.85	8.45–11.47	1.24	0.99–1.54
Whole milk of own/local production	No	8.64	7.76–9.61	1 (reference)	
	Yes	18.25	9.90–33.64	2.11	1.13–3.94
Fruit/Vegetables of own/local production	No	8.06	7.05–9.23	1 (reference)	
	Yes	11.03	8.80–13.82	1.37	1.03–1.82

Notes: GM exp.: expected geometric mean estimated by regression model; 90% CI: confidence interval at 90% probability; GMR: geometric mean ratio.

**Table 4:** Amiata sample. Factors associated with u(iAs+MMA+DMA) concentration by stepwise multivariate regression.

Factors Selected (*p* < 0.2)	Class	GM Exp.	90% CI	GMR	90% CI
GSTT	-	5.57	3.72–8.32	1 (reference)	
	+	3.21	2.54–4.06	0.58	0.36–0.91
Smoker	No	3.10	2.44–3.94	1 (reference)	
	Yes	4.97	3.43–7.22	1.61	1.04–2.48
Seafood consumption 3 days before urine collection	No	3.02	2.40–3.81	1 (reference)	
	Yes	6.56	4.37–9.84	2.17	1.37–3.43
Meat	No	2.85	2.07–3.91	1 (reference)	
	Yes	4.62	3.55–6.00	1.62	1.07–2.45
Whole milk of own/local production	No	3.51	2.85–4.34	1 (reference)	
	Yes	9.17	5.13–16.38	2.61	1.42–4.79

Notes: GM exp.: expected geometric mean estimated by regression model; 90% CI: confidence interval at 90% probability; GMR: geometric mean ratio.

**Table 5:** Viterbese sample. Factors associated with u(iAs+MMA+DMA) concentration by stepwise multivariate regression.

Factors Selected (*p* < 0.2)	Class	GM Exp.	90% CI	GMR	90% CI
Tap water	No	6.85	5.41–8.68	1 (reference)	
	Yes	9.23	7.53–11.31	1.35	1.02–1.77
Smoker	No	7.50	6.15–9.16	1 (reference)	
	Yes	10.55	8.24–13.51	1.41	1.06–1.86
Exposure to inorganic solvents and acids	No	6.90	5.93–8.02	1 (reference)	
	Yes	19.42	9.89–38.14	2.81	1.40–5.67
Whole milk	No	7.77	6.42–9.39	1 (reference)	
	Yes	10.75	8.23–14.04	1.38	1.04–1.84
Seafood consumption 3 days before urine collection	No	6.46	5.29–7.88	1 (reference)	
	Yes	19.75	15.18–25.70	3.06	2.25–4.16

Notes: GM exp.: expected geometric mean estimated by regression model; 90% CI: confidence interval at 90% probability; GMR: geometric mean ratio.

**Table 6:** Taranto sample. Factors associated with u(iAs+MMA+DMA) concentration by stepwise multivariate regression.

Factors Selected (*p* < 0.2)	Class	GM Exp.	90% CI	GMR	90% CI
AS3MT	No	5.64	3.59–8.87	1 (reference)	
	Yes	8.19	4.84–13.87	1.45	0.92–2.30
Tap water	No	3.48	1.43–8.47	1 (reference)	
	Yes	8.29	5.66–12.13	2.38	0.99–5.73
Exposure to inorganic solvents and acids	No	5.17	3.31–8.07	1 (reference)	
	Yes	14.81	8.28–26.50	2.86	1.69–4.84
Seafood	No	3.56	1.78–7.12	1 (reference)	
	Yes	7.34	4.83–11.15	2.06	1.15–3.71
Meat	No	4.78	3.20–7.14	1 (reference)	
	Yes	8.31	4.73–14.61	1.74	1.07–2.81
Bread, pasta, cereals	No	1.85	0.64–5.39	1 (reference)	
	Yes	6.61	4.34–10.08	3.57	1.40–9.10
Whole milk	No	4.81	3.11–7.45	1 (reference)	
	Yes	14.75	7.99–27.23	3.06	1.78–5.26
Coffee	No	3.43	1.97–5.97	1 (reference)	
	Yes	7.64	4.81–12.14	2.23	1.29–3.84
Fruit/Vegetables of own/local production	No	4.95	2.87–8.53	1 (reference)	
	Yes	11.17	7.92–15.77	2.26	1.31–3.88

Notes: GM exp.: expected geometric mean estimated by regression model; 90% CI: confidence interval at 90% probability; GMR: geometric mean ratio.

**Table 7:** Gela sample. Factors associated with u(iAs+MMA+DMA) concentration by stepwise multivariate regression.

Factors Selected (*p* < 0.2)	Class	GM Exp.	90% CI	GMR	90% CI
GSTT	-	22.43	14.12–35.62	1 (reference)	
	+	9.68	7.65–12.25	0.43	0.26–0.72
Occupational exposure in chemical industrials	No	10.48	8.34–13.17	1 (reference)	
	Yes	36.67	23.15–58.08	3.50	2.09–5.85
Seafood	No	6.89	4.55–10.44	1 (reference)	
	Yes	13.37	10.48–17.04	1.94	1.21–3.12
Coffee	No	19.86	13.45–29.31	1 (reference)	
	Yes	9.81	7.66–12.57	0.49	0.31–0.78

Notes: GM exp.: expected geometric mean estimated by regression model; 90% CI: confidence interval at 90% probability; GMR: geometric mean ratio.

**Table 8:** Geometric mean, 50p and 95p values for u(iAs+MMA+DMA) concentration in our study and in other studies reporting reference values.

Country	Acronym of the Study	Year of Recruitment	Age Class	*n*	GM	50p	95p	Reference
Italy	SEpiAs	2010	20–44	271	8.76	7.78	86.28	[49]
Germany	GerES-III	1998	18–69 25–69	4741 4052	3.92 3.87	4.1 4.0	18.9 19.3	[90,91]
France	ENNS	2006–2007	18–39 18–74	444 1500	4.07 3.75	4.49 4.03	10.72 10.68	[92]
France	ENNS	2006–2007	18–74	3015	3.75	nr	nr	[83]
USA	NHANES	2009–2010 2011–2012	>20 years	2020 1724	6.7 5.6	5.95 5.15	23.2 17.6	[57]

Notes—*n*: sample size; nr: not reported; 50p: 50th percentile; 95p: 95th percentile; GerES-III: third German Environmental Surveys; ENNS: French Nutrition and Health Service; NHANES: National Health and Nutrition Examination Survey.

## Supplementary Materials

The following are available online at http://www.mdpi.com/1660-4601/15/2/299/s1, Table S1: Overall sample. Factors associated with uiAs concentration by stepwise multivariate regression analysis, Table S2: Overall sample. Factors associated with ui(MMA+DMA) concentration by stepwise multivariate regression analysis.
