# Supplementary material for: Urinary Arsenic in Human Samples from Areas Characterized by Natural or Anthropogenic Pollution in Italy
Source: Int J Environ Res Public Health. 2018 Feb 9;15(2):299. doi: 10.3390/ijerph15020299 (PMC5858368; doi:10.3390/ijerph15020299)
Supplement: Supplementary file 1 [file ijerph-15-00299-s001.docx]

**Table S1.** Overall sample. Factors associated with uiAs concentration by stepwise multivariate regression analysis.

| **Factors Selected (*p* < 0.2)** | **Class** | **GM Exp** | **90%CI** | **GMR** | **90%CI** |
| --- | --- | --- | --- | --- | --- |
| Area | Amiata | 0.79 | 0.6–1.03 | 1 (reference) |  |
|  | Viterbese | 2.21 | 1.72–2.83 | 2.59 | 1.8–3.73 |
|  | Taranto | 2.62 | 1.88–3.65 | 3.18 | 2.03–4.97 |
|  | Gela | 3.67 | 2.93–4.61 | 4.36 | 3.03–6.28 |
| GSTT | - | 3.15 | 2.42–4.09 | 1 (reference) |  |
|  | + | 1.93 | 1.68–2.21 | 0.62 | 0.46–0.84 |
| Occupational exposure in chemical industrial | no | 2.02 | 1.78–2.3 | 1 (reference) |  |
|  | yes | 4.21 | 2.68–6.61 | 2.11 | 1.31–3.38 |
| Exposure to inorganic solvents and acids | no | 1.99 | 1.74–2.28 | 1 (reference) |  |
|  | yes | 2.99 | 2.21–4.03 | 1.49 | 1.07–2.09 |
| Seafood | no | 1.47 | 1.12–1.92 | 1 (reference) |  |
|  | yes | 2.38 | 2.08–2.72 | 1.59 | 1.17–2.15 |
| Seafood_3 days | no | 1.81 | 1.58–2.08 | 1 (reference) |  |
|  | yes | 3.76 | 2.92–4.84 | 2.06 | 1.54–2.76 |
| Whole milk | no | 1.94 | 1.69–2.23 | 1 (reference) |  |
|  | yes | 2.94 | 2.29–3.78 | 1.55 | 1.16–2.07 |
| Meat | no | 1.87 | 1.57–2.22 | 1 (reference) |  |
|  | yes | 2.47 | 2.08–2.94 | 1.35 | 1.05–1.73 |
| Whole milk own/local production | no | 8.64 | 7.76–9.61 | 1 (reference) |  |
|  | yes | 18.25 | 9.9–33.64 | 3.40 | 1.61–7.19 |
| Fruit-Vegetables own/local production | no | 1.94 | 1.67–2.27 | 1 (reference) |  |
|  | yes | 2.72 | 2.1–3.52 | 1.54 | 1.09–2.17 |

**Table S2.** Overall sample. Factors associated with ui(MMA+DMA) concentration by stepwise multivariate regression analysis.

| **Factors Selected (*p* < 0.2)** | **Class** | **GM Exp** | **90%CI** | **GMR** | **90%CI** |
| --- | --- | --- | --- | --- | --- |
| Area | Amiata | 3.01 | 2.41–3.75 | 1 (reference) |  |
|  | Viterbese | 5.89 | 4.82–7.21 | 1.96 | 1.46–2.64 |
|  | Taranto | 8.15 | 6.22–10.67 | 2.71 | 1.9–3.88 |
|  | Gela | 9.47 | 7.88–11.39 | 3.15 | 2.35–4.23 |
| GSTT | - | 8.04 | 6.48–9.98 | 1 (reference) |  |
|  | + | 5.90 | 5.27–6.6 | 0.73 | 0.57–0.94 |
| Occupational exposure in chemical industrials | no | 5.80 | 5.23–6.43 | 1 (reference) |  |
|  | yes | 16.23 | 11.33–23.25 | 2.80 | 1.92–4.08 |
| Seafood | no | 4.97 | 3.99–6.2 | 1 (reference) |  |
|  | yes | 6.73 | 6.01–7.52 | 1.35 | 1.05–1.74 |
| Seafood_3 days | no | 5.62 | 5.01–6.29 | 1 (reference) |  |
|  | yes | 9.23 | 7.51–11.36 | 1.64 | 1.3–2.09 |
| Whole milk | no | 5.95 | 5.31–6.67 | 1 (reference) |  |
|  | yes | 7.59 | 6.17–9.32 | 1.27 | 1–1.62 |
| Meat | no | 5.73 | 4.97–6.61 | 1 (reference) |  |
|  | yes | 6.95 | 6.02–8.02 | 1.21 | 0.99–1.49 |
| Whole milk own/local production | no | 6.17 | 5.58–6.83 | 1 (reference) |  |
|  | yes | 12.26 | 6.9–21.8 | 1.99 | 1.1–3.57 |
| Fruit-Vegetables own/local production | no | 5.89 | 5.19–6.68 | 1 (reference) |  |
|  | yes | 7.44 | 6.02–9.2 | 1.26 | 0.97–1.65 |
